# Clinical parameters predict the effect of bilateral subthalamic stimulation on dynamic balance parameters during gait in Parkinson's disease

**DOI:** 10.3389/fneur.2022.917187

**Published:** 2022-09-26

**Authors:** Andrea Kelemen, László Halász, Muthuraman Muthuraman, Loránd Erőss, Péter Barsi, Dénes Zádori, Bence Laczó, Dávid Kis, Péter Klivényi, Gábor Fekete, László Bognár, Dániel Bereczki, Gertrúd Tamás

**Affiliations:** ^1^Department of Neurology, Semmelweis University, Budapest, Hungary; ^2^National Institute of Clinical Neurosciences, Budapest, Hungary; ^3^Biomedical Statistics and Multimodal Signal Processing Unit, Department of Neurology, University Medical Center of Johannes Gutenberg University Mainz, Mainz, Germany; ^4^Department of Neuroradiology, Medical Imaging Centre, Semmelweis University, Budapest, Hungary; ^5^Department of Neurology, University of Szeged, Szeged, Hungary; ^6^Department of Neurosurgery, University of Szeged, Szeged, Hungary; ^7^Department of Neurosurgery, University of Debrecen, Debrecen, Hungary

**Keywords:** Parkinson's disease, dynamic balance, ITUG, subthalamic nucleus, deep brain stimulation, double support, gait, sway

## Abstract

We investigated the effect of deep brain stimulation on dynamic balance during gait in Parkinson's disease with motion sensor measurements and predicted their values from disease-related factors. We recruited twenty patients with Parkinson's disease treated with bilateral subthalamic stimulation for at least 12 months and 24 healthy controls. Six monitors with three-dimensional gyroscopes and accelerometers were placed on the chest, the lumbar region, the two wrists, and the shins. Patients performed the instrumented Timed Up and Go test in stimulation OFF, stimulation ON, and right- and left-sided stimulation ON conditions. Gait parameters and dynamic balance parameters such as double support, peak turn velocity, and the trunk's range of motion and velocity in three dimensions were analyzed. Age, disease duration, the time elapsed after implantation, the Hoehn-Yahr stage before and after the operation, the levodopa, and stimulation responsiveness were reported. We individually calculated the distance values of stimulation locations from the subthalamic motor center in three dimensions. Sway values of static balance were collected. We compared the gait parameters in the OFF and stimulation ON states and controls. With cluster analysis and a machine-learning-based multiple regression method, we explored the predictive clinical factors for each dynamic balance parameter (with age as a confounder). The arm movements improved the most among gait parameters due to stimulation and the horizontal and sagittal trunk movements. Double support did not change after switching on the stimulation on the group level and did not differ from control values. Individual changes in double support and horizontal range of trunk motion due to stimulation could be predicted from the most disease-related factors and the severity of the disease; the latter also from the stimulation-related changes in the static balance parameters. Physiotherapy should focus on double support and horizontal trunk movements when treating patients with subthalamic deep brain stimulation.

## Introduction

The effect of subthalamic deep brain stimulation (STN-DBS) on dynamic balance during gait in Parkinson's disease (PD) has not yet been investigated in detail. Dynamic balance during a movement, e.g., while walking, is one component of the complex balance process, in addition to balance during quiet stance, reactive postural adjustment to external perturbations, and anticipatory postural adjustment in preparation for voluntary movements (1).

The dynamic imbalance in PD derives from several elements. First, it depends on the gait abnormalities characteristic of the disease stage. Reductions in step length and gait speed, reduced swinging of the arms, and increased interlimb asymmetry are frequently reported at the early stage, while turning deficits, gait initiation difficulty, and freezing develop at the mild-to-moderate stage, as well as further gait irregularities due to motor fluctuations and dyskinesias at the advanced stage (2). Second, other disease-related factors were shown to influence dynamic balance during walking, such as even subclinical cognitive impairment, executive dysfunction (3), and fear of falling (1). Although levodopa treatment improves gait speed, facilitates step initiation and anticipatory postural adjustment (4), and reduces gait variability (5), it also raises sway during stance in PD (6). Apparent cholinergic dysfunction was revealed in levodopa-resistant gait abnormalities (7). Third, age is an additive risk factor for poor postural control (8).

The positive effect of STN-DBS on balance tends to taper off after the first nine postoperative months (9). Gait parameters also improve in the first 10 months, especially when STN-DBS is combined with levodopa therapy (10, 11), but deteriorate 3 years after the operation (12, 13).

There is a lack of information about the impact of the STN-DBS on dynamic balance during natural walking. The widely used clinical scales do not assess the different features of gait and balance separately in PD (14) for exploring stimulation-related disturbances (15, 16). Posturography (6, 9, 17) or motion sensor studies (18) on DBS either investigated quiet stances in single (6, 9, 19) or dual-task conditions (6, 17). Gait parameters were analyzed separately with scales (9, 20) or motion sensors (16, 21). The Timed Up and Go test complements the gait analysis by detecting turning and postural transitions, and its total duration time is well correlated to fall risk (22). Its advanced version, the Instrumented Timed Up and Go (ITUG) test, utilizes motion sensors and provides sensitive and reliable gait parameters correlating with the Unified Parkinson's Disease Rating Scale (UPDRS) motor scores (23).

Therefore, we aimed to observe the more than 1-year-long bilateral STN-DBS effect on gait and turning parameters by focusing on the dynamic balance. We hypothesized that gait parameters do not improve with stimulation to the normal control level and that there is a relationship between the stimulation-induced changes in dynamic balance and the disease-related clinical parameters and electrode localization. We also assumed that stimulation-induced changes in dynamic balance during gait are not independent of the postural sway during quiet stance and are measured in sensory conflict situations.

## Materials and methods

### Participants

We recruited 24 patients with PD treated with bilateral STN-DBS and an age-matched group of 24 healthy controls. The Core Assessment Program for Surgical Interventional Therapies for Parkinson's Disease (24) was followed when indicating the surgery. The inclusion criteria of the patients were as follows: at least 12 months had passed since the operation, stable stimulation parameters, and clinical state for at least 3 months. Exclusion criteria were significant orthopedical/rheumatological disorders or visual disability not correctable with eyeglasses. We excluded four patients because they could not walk in the medication and stimulation OFF state. Finally, 20 patients completed the tasks, and none had levodopa-resistant freezing.

For individual anatomical planning of the surgery, preoperative contrast-enhanced MR (3T Philips Achieva) images and stereotactic contrast-enhanced CT sequences (made on the day of surgery) were merged using the Medtronic FrameLink 5 software. Intraoperative electrophysiological mapping was executed with five microelectrodes; macro stimulation controlled clinical symptoms (25).

Ethical approval (reference number: 271/2013) was obtained from the Regional and Institutional Committee of Science and Research Ethics, Semmelweis University, and patients signed informed consent forms.

### Measurement protocol

**Figure 1:**
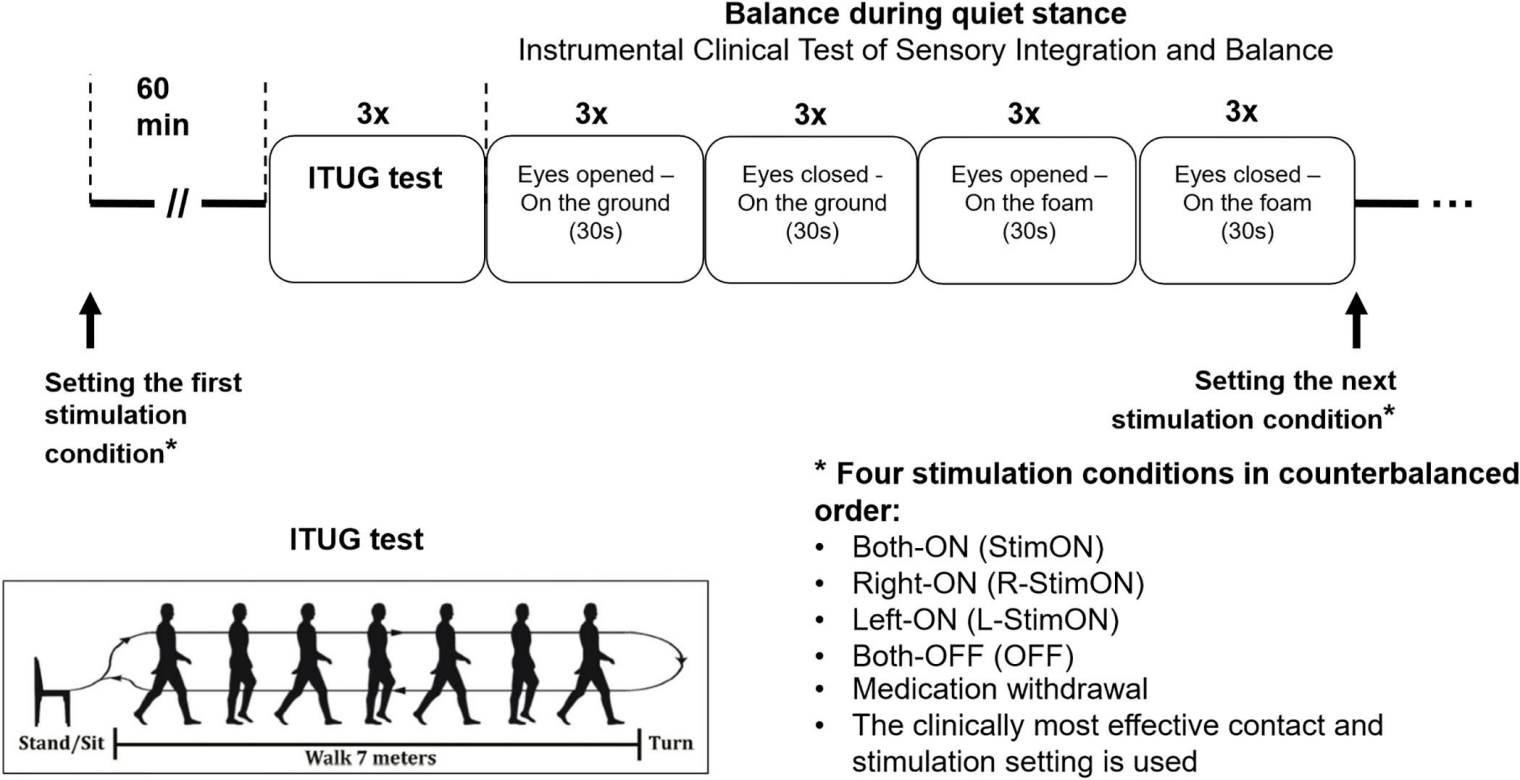
Study protocol.

Six wireless Opal monitors (APDM Inc.) (18) consisting of three-dimensional gyroscopes and accelerometers were placed on the chest, the lumbar region, the wrists, and the shins (Figure 1). The sample rate was 128 Hz. The subjects executed the ITUG test with the four major components: sit-to-stand, 7 m long gait, turning, and turn-to-sit tasks (Figure 1). At the beginning of the test, the subject sat on a chair (without an armrest) with their hands placed on their knees. After a sound cue, the patient stood up without using the arms, walked 7 m with a dynamic walking speed until reaching the target line, then turned back and walked back to the chair. Finally, the subject sat back and put their hands on their knees again (23). The average values of three consecutive trials were further analyzed to increase reliability.

We assessed the balance during quiet stance with the Instrumental Clinical Test of Sensory Integration and Balance (ICTSIB) with the following parts: stance on the plain ground with arms folded across the chest with eyes open and closed and stance on foam with arms folded across the chest with eyes open and closed (26).

The patients were on at least a 12-h medication withdrawal before the measurements. They then repeated the ITUG and ICTSIB tasks in four stimulation conditions: bilateral stimulation OFF (OFF), bilateral stimulation ON (StimON), unilateral right-sided (R-StimON), and left-sided (L-StimON) stimulation ON, in counterbalanced order. We stimulated the clinically used contacts during the study, with the stimulation parameters used for therapeutic purposes. A 1-h time interval was maintained as a washout period between testing in two different stimulation conditions. Healthy controls also executed the ITUG and ICTSIB tests three times in one session for averaging. The measurement protocol is presented in Figure 1.

### The outcome measures

The ITUG gait parameters were collected and calculated by the Mobility Lab Software (APDM Inc.). We compared the values measured in the four stimulation conditions with the values of the control group, calculated the StimON/OFF improvement, and compared the StimON and OFF parameters in the PD group (Supplementary Table 1). We chose the potential indicator parameters of the dynamic balance as follows: double support (percentage of the gait cycle time that both feet are on the ground, where the gait cycle means the period between two consecutive heel-strikes of the right foot); peak turning velocity (peak angular velocity of the trunk during turning), the range of motion (ROM, degree), as well as velocity (degree/s) of the trunk in the horizontal, sagittal, and frontal plane (27).

### The potential predictors of clinical factors

We collected the following disease-related parameters: age, disease duration, the pre-and postoperative Hoehn-Yahr stage, the time elapsed since the operation, and the levodopa responsiveness calculated from the rate of UPDRS III scores in preoperative MED ON and OFF states (dopamine agonists were only stopped 1 day before the test because patients did not tolerate the discomfort). In addition, we determined the International Parkinson and Movement Disorders Society MDS-UPDRS III scores in the StimON and OFF stimulation conditions and their ratio to the stimulation responsiveness at the time of measurement.

We collected the sway values (m^2^/s^4^; the area of the 95% confidence ellipse, an average of the three trials) in the four tasks of the ICTSIB test; their average as combined sway (18, 26) was subsequently used among the potential clinical predictors.

We specified the anatomical location of the active contacts as described in Kelemen et al. (26) in detail. In short, the postoperative CT scans acquired at least 3 months after lead implantation were co-registered with anatomical T1 images. The coordinates of the active contacts were calculated using Euclidean vectorial calculations; the reference point was the mathematical center point of the dorsolateral motor portion of the STN, according to Atlas (28). Distances between the active contacts and the warped motor centers were calculated in each plane and three dimensions in millimeters.

### Statistical analyses

The normal distribution of the data was first determined with the Kolmogorov-Smirnov test; according to the results, we used parametric or nonparametric statistical tests. The age of the PD and control group was compared with the Mann–Whitney *U* test. The active contact locations referenced to the center of the dorsolateral STN and the stimulation intensity on the left and right sides were compared with an unpaired Student *t*-test. The parameters of the ITUG test in the different stimulation conditions were compared with control values using the unpaired Student *t*-test; the *p*-value was determined after a Bonferroni correction. Finally, the parameters in the stimulation conditions were compared with ANOVA for repeated measures within the PD group. The determining factor was the STIMULATION CONDITION; we used Tukey's test for multiple comparisons. The level of significance was set at *p* < 0.05.

### Support vector regression analyses

We performed a support vector regression (SVR) analysis—representing a machine-learning-based multiple regression method—that could associate the observed and trained values and present the regression coefficient for prediction accuracy (29). This study implemented a data-driven regression model without explicitly stating a functional form, indicating a non-parametric technique.

In short, the algorithm looks for an optimally separating threshold between the two data sets by maximizing the margin between the classes' closest points. The points lying on the boundaries are called support vectors, and the middle of the margin is the optimal separating threshold. Since, in most cases, using a linear separator is not ideal, a projection into a higher-dimensional space was performed, whereby the data points effectively become linearly interrelated. Here, we have used the radial basis function kernel for this projection due to its good performance, as discussed in (30), and used the grid search (min = 1; max = 10) to find the few optimal input parameters, namely, *R* (type of regression algorithm; 1–1,000) and gamma (0.25). A soft-margin classifier of the calculated independent variables was used for every parameter, and a penalty constant *P* weighted spurious correlations. In order to optimize regression accuracy, this was calculated for every regressor. We performed the following steps to demonstrate that no overfitting was attested in our data for the SVR regression algorithm. The results from the SVR are reported here with fivefold cross-validation. Additionally, we used age as a confound in the analyses. We used 70% of the data for training and 30% of the data for testing.

## Results

### Demographics and clinical parameters

The characteristics of the patient group are summarized in Table 1. The age (median/IQR) of the patients (63/58–68.5 years) and the controls (58/52.3–69 years) did not differ (*p* = 0.46). Five females and 15 males were in the PD group, and 13 females and 11 males were in the control group. The MDS-UPDRS 3.11 scores (freezing of gait) improved in one patient from 3 to 1 while turning the bilateral stimulation on and remained 1 in one patient. No freezing of gait was observed in OFF and StimOn conditions in the rest of the patient group. In the OFF state, four patients had a score of 3, two patients had a score of 2, and four patients had a score of 1 on the MDS-UPDRS 3.12 scale representing postural stability. All other patients had a score of 0. The latest scores improved in five patients and worsened in four patients after switching on the bilateral stimulation.

**Table 1:** Demographics and clinical data of the patients.

**Feature**		**Values; median (IQR)**
Disease duration at the time of surgery		11 (9.5–14) years
Time since surgery		19 (13.5–40) months
Levodopa equivalent dose	Preoperative	816 (588–931) mg
	At the study	266 (200–586) mg
Preoperative UPDRS III. score	MED-OFF	29 (23–51) points
	MED-ON	6 (1–11) points
MDS-UPDRS III. score at the study	MED-OFF, BOTH-OFF	37 (22.5–47) points
	MED-OFF, BOTH-ON	15 (7–19) points
Levodopa response	Preoperative	86 (77–100) %
Stimulation response	At the study	65 (50–71) %
Hoehn-Yahr stage	Preoperative	3 (2.5–3)
	1 year after the operation	1 (1–1.5)

### The location of the active contacts and parameters of the stimulation

The active contact locations and the stimulation parameters are presented in Table 2. The active contact locations in the three planes, right and left (*x*: *p* = 0.28; *y*: *p* = 0.8; *z*: *p* = 0.36), did not differ. There was no significant difference in the stimulation intensity on the two sides (*p* = 0.36).

**Table 2:** Parameters of the stimulation and distance of the active contact from the motor center of the dorsolateral STN.

**Feature**		**Right**	**Left**
STN stimulation	Amplitude (V; mean ± SD)	2.3 ± 0.8	2.4 ± 2.65
	Frequency (Hz; median and IQR)	130 (130–145)	130 (130–145)
	Impulse width (μs; median and IQR)	60 (60–65)	60 (60–65)
Location distance from center of dorsal STN (mm; mean ± SD)	X	0.46 ± 1.97	0.55 ± 0.34
	Y	−1.44 ± 1.58	−1.24 ± 0.33
	Z	0.48 ± 0.43	0.51 ± 0.53

### Comparison of the ITUG parameters between PD and the control group

The PD group performed worse than controls both in OFF and StimON conditions regarding the following parameters: total duration of the ITUG Test, turn duration, and turn-to-sit duration (Supplementary Table 1).

### Effect of bilateral subthalamic stimulation on the parameters of the ITUG test

**Figure 2:**
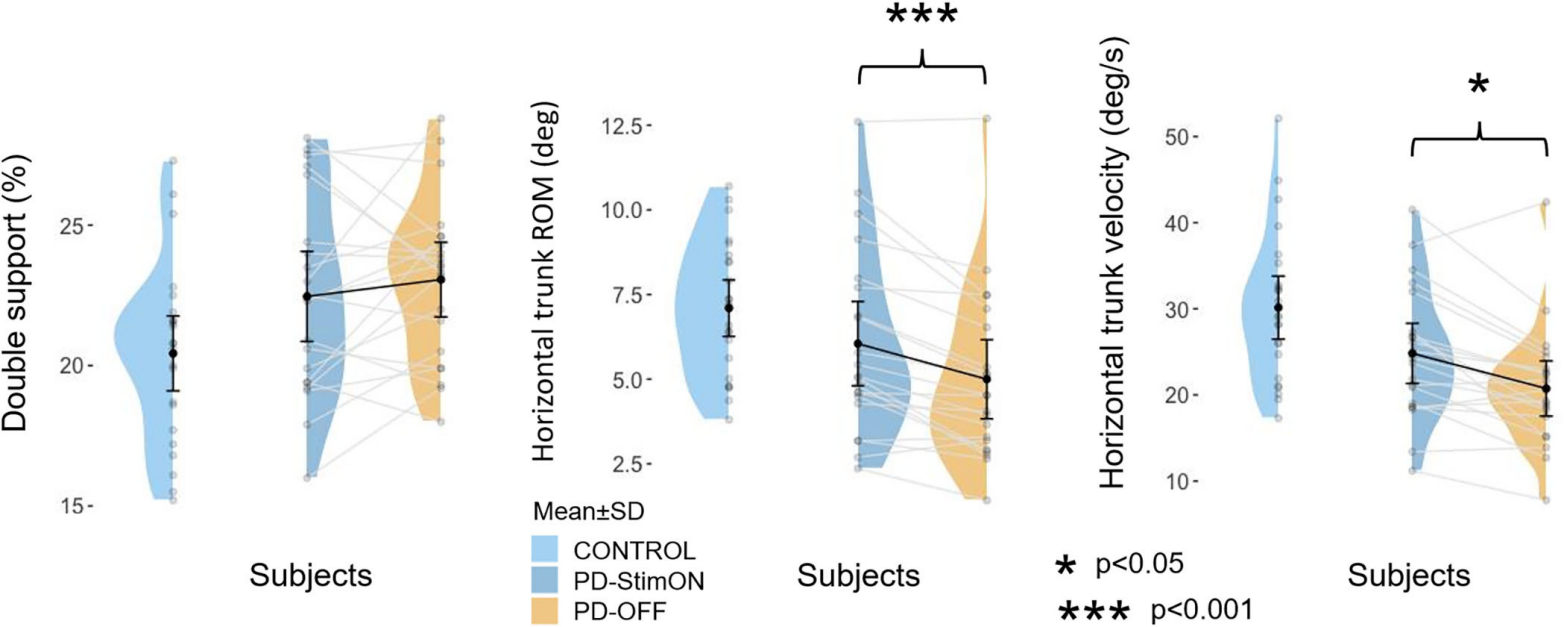
Double support and horizontal trunk movements in the patient and the control group. Improvement of double support is individual in the PD group, whereas horizontal trunk range of motion and velocity significantly ameliorates when switching the stimulation on.

The majority of the measured parameters were significantly improved by bilateral subthalamic stimulation. The ROM of the left and right arms improved the most in StimON compared to the OFF condition, followed by the arm's velocities and the trunk's ROM and velocity in the horizontal and sagittal planes (Figure 2, Supplementary Table 1). Turning on the stimulation did not affect the double support at the group level, which was not different from the control values.

### Individual changes in the parameters of dynamic balance due to stimulation

Double support improved, decreased, or did not worsen in 10 of the 20 patients. In comparison, changes in other parameters were more homogenous (improvement/increase in turn peak velocity: 17/20 patients, trunk ROM horizontal: 17/20 patients, sagittal: 14/20 patients, frontal: 15/20 patients; trunk velocity horizontal: 15/20 patients, sagittal: 19/20 patients, frontal: 16/20 patients).

### Prediction analysis of the parameters of dynamic balance

Table 3 presents the accuracy of how the clinical factors predicted the stimulation-induced changes in dynamic balance parameters. The superior-inferior and the anterior-posterior deviations from the motor center of the STN predicted the improvement rate of most parameters. The more posterior and inferior locations in the dorsolateral area improved the dynamic balance. In addition, the changes in the horizontal trunk movements could be predicted from the disease duration, stimulation responsiveness, and the stimulation-induced improvement of the combined postural sway from the ICTSIB test. Stimulation-induced alterations of the double support and the horizontal ROM could be predicted from the largest number of disease-related factors and the active contact location (Table 3). Improvement of double support was associated with the severity of the motor symptoms and the stimulation-induced quiet stance imbalance, in addition to anterior-posterior and superior-inferior contact locations.

**Table 3:** Coefficients of the support vector regression (SVR) analysis.

	**StimON/OFF ratio**							
	**Double support**	**Turn peak velocity**	**Trunk ROM**			**Trunk velocity**		
			**Horizontal**	**Sagittal**	**Frontal**	**Horizontal**	**Sagittal**	**Frontal**
Disease duration	0.55	0.52	0.75*	0.58	0.48	0.80*	0.76*	0.68
PreOP levodopa response	0.54	0.52	0.65	0.61	0.64	0.67	0.64	0.62
Stimulation response	0.55	0.58	0.75*	0.57	0.64	0.62	0.62	0.63
MDS-UPDRS III. MED OFF, Stim OFF	0.71*	0.57	0.55	0.57	0.44	0.59	0.47	0.38
StimON/OFF Combined sway	0.78*	0.65	0.42	0.41	0.55	0.71*	0.43	0.47
RX	0.47	0.45	0.42	0.41	0.55	0.46	0.44	0.48
RY	0.75*	0.76*	0.65	0.66	0.55	0.62	0.80*	0.46
RZ	0.72*	0.56	0.73*	0.47	0.72*	0.75*	0.48	0.72*
LX	0.58	0.48	0.47	0.46	0.74*	0.54	0.76*	0.45
LY	0.54	0.70*	0.82*	0.47	0.85*	0.48	0.46	0.82*
LZ	0.66	0.47	0.43	0.42	0.68	0.45	0.42	0.48

Significant predictions are marked with an asterisk.

ROM, range of motion; R, right; L, left; y, anterior (–)-posterior (+), z, superior (+)-inferior (–), x, medial (right + left-)-lateral (right-left+).

## Discussion

With our results, we show that highlighted parameters of dynamic balance improve after switching on the STN stimulation, such as trunk range of motion, velocity in the three dimensions, and the turn peak velocity in the ITUG test; however, their values do not achieve the level of the control values. The double support improved the least and did not differ between the OFF and StimON conditions and controls. Its value was individually variable and could be predicted by the absence of medication and off-stimulation motor symptoms. The double support, the horizontal ROM, and the trunk velocity could be predicted by clinical factors that represented the state of the disease, such as the disease duration and postural stance imbalance. We also showed that upper limb movements improved the most with STN-DBS among the ITUG parameters.

### Double support

Double support is the percentage of the gait cycle time that both feet are on the ground (27); it is associated with the freezing of gait in PD (7). Levodopa has a positive effect (31, 32) or no effect (33, 34) on double support during both short-term [3–6 months; (31) vs. (34)] and long-term [10–39 months; (32) vs. (33, 35), sequentially] STN-DBS treatment. A combined intervention of medication and stimulation was shown to exert a better effect on the freezing of gait than either treatment alone in a 6–12-months follow-up period (11). Our study has pinpointed that its stimulation-induced change is individually variable and determined by disease-related factors. Results from a large cohort of PD patients (331 patients) support our findings according to which the outcome of STN-DBS on the freezing of gait relates to the severity of the symptoms in the preoperative phase, the severity of the motor fluctuations, the brain atrophy, and the postoperative cognitive performance (36). DBS modulates targeted, selected brain networks, in which dopamine plays a key role (37). In contrast, freezing and falls were associated with cholinergic dysfunction involving the brainstem pedunculopontine nucleus (38), which explains the insufficient effect of STN-DBS treatment on these symptoms. Non-levodopa-responsive axial symptoms appear along with the disease progression (39).

### Horizontal trunk movements

This study analyzed trunk movement in three dimensions, showing that the clinical state most influences the horizontal plane's motions. Accordingly, it was reported that the mediolateral sway area during quiet stance is more affected in PD than the anteroposterior, even in the early phase of the disease (40). Levodopa therapy worsens this abnormality, whereas STN-DBS reduces it and stabilizes balance in combination therapy (6, 40). We report for the first time that the stimulation-induced improvement during ITUG can be predicted from the disease duration and the stimulation responsiveness. Besides that, stimulation-induced changes in horizontal trunk velocity could be significantly predicted from the stimulation-induced combined static sway according to their interrelation in the complex balance function (1). Our results confirm that the mediolateral sway is disease-specific (1) and that the disease progression influences the DBS effect on it. It may cause a tendency to fall in the mediolateral direction in PD.

### Effect of the active contact location

Dynamic balance could also be predicted from the active contact location in our study. A more superior location on the right side predicted less stimulation-induced improvement of double support and trunk movement range and velocity in the horizontal and frontal plane. The more posterior location was beneficial for most parameters of the dynamic balance. It was earlier demonstrated that high-frequency stimulation of the pedunculopontine nucleus worsens axial symptoms (41). The anatomical arrangement of its associative pathways explains our experiences while stimulating the dorsolateral STN. Ventral STN stimulation impairs gait (42). Stimulation anterodorsal from the STN may reach the Forel's field H2 with the passing pallido-pedunculopontine fibers, resulting in gait disturbances (20, 36).

### Effect of STN-DBS on other gait parameters

Stride velocity improved significantly by STN-DBS in agreement with former studies (43), as well as trunk velocity in the three dimensions, the turn peak velocity, the sit-to-stand velocity, and the turn-to-sit velocity. The stride length, the range of motion of the trunk in three dimensions, and the sit-to-stand position transition have also been raised as expected (10). In contrast, the temporal parameters such as cadence and gait cycle time were not influenced by switching the stimulation on, similarly to earlier results (7). Our results confirm that DBS acts more on appendicular than axial movements (42) as the arms' range of motion is most elevated.

### Strengths and limitations of the study

The study's strengths include using objective motion analysis to describe gait and dynamic balance. We also measured the anatomical location of the active contacts among the clinical characteristics.

A limitation is the number of recruited patients; it would be beneficial to perform the study in a larger cohort. Furthermore, a more extended washout period between stimulation conditions might be ideal for testing axial symptoms (44). Although, during stimulation OFF or unilateral stimulation, patients feel discomfort and cannot be burdened with this state for hours.

Furthermore, we have used a data-driven machine learning approach in this study, so caution needs to be taken in interpreting the results. The small sample size with the multiple parameter space limits the external validity and needs to be replicated in other centers and larger cohorts of patients.

### Conclusion

The improvement of the double support and the horizontal trunk movements by STN-DBS are most affected by disease-related factors. Therefore, these symptoms should be focused on by the physiotherapy of patients with STN-DBS. The detailed kinematic analysis provides new information to plan an appropriate multidisciplinary approach for patient management after DBS implantation.

## Data availability statement

The original contributions presented in the study are included in the article/Supplementary material, further inquiries can be directed to the corresponding author.

## Ethics statement

The studies involving human participants were reviewed and approved by Regional and Institutional Committee of Science and Research Ethics, Semmelweis University (reference number: 271/2013). The patients/participants provided their written informed consent to participate in this study.

## Author contributions

AK, LH, MM, and GT: conception and design of the work. AK, LH, LE, PB, DZ, BL, DK, PK, GF, and GT: acquisition. AK, LH, MM, and GT: analysis or interpretation of data for the work. AK, LH, MM, DZ, LB, DB, and GT: drafting the work or revising it critically for important intellectual content. AK, LH, MM, LE, PB, DZ, BL, DK, PK, GF, LB, DB, and GT: provide approval for publication of the content and agree to be accountable for all aspects of the work in ensuring that questions related to the accuracy or integrity of any part of the work are appropriately investigated and resolved. All authors contributed to the article and approved the submitted version.

## Conflict of interest

The authors declare that the research was conducted without any commercial or financial relationships that could be construed as a potential conflict of interest.

## Publisher's note

All claims expressed in this article are solely those of the authors and do not necessarily represent those of their affiliated organizations, or those of the publisher, the editors and the reviewers. Any product that may be evaluated in this article, or claim that may be made by its manufacturer, is not guaranteed or endorsed by the publisher.

## Supplementary material

Supplementary Table 1 ITUG parameters in the different stimulation conditions in the PD group and controls.

## Abbreviations

ITUG: instrumented timed up and go test
ICTSIB: instrumental clinical test of sensory integration and balance
ROM: range of motion
SVR: support vector regression (SVR) analysis.
