# Supplementary material for: Clinical parameters predict the effect of bilateral subthalamic stimulation on dynamic balance parameters during gait in Parkinson's disease
Source: Front Neurol. 2022 Sep 26;13:917187. doi: 10.3389/fneur.2022.917187 (PMC9549153; doi:10.3389/fneur.2022.917187)
Supplement: Supplementary Table 1 — ITUG parameters in the different stimulation conditions in the PD group and controls. [file Table_1.docx]

**Supplementary Table 1.** ITUG parameters in the different stimulation conditions in the PD group; and in controls

| **Parameters** | **OFF** | **StimON** | **R-StimON** | **L-StimON** | **Control** | **StimON/OFF**  **improvement (%)** | **StimON-OFF p** |
| --- | --- | --- | --- | --- | --- | --- | --- |
|  | (mean±SD; *p<0.05 in comparison with control values) | | | | | |  |
| **ITUG total duration** (s) | 22.7±5.80* | 20.3±3.89* | 20.8±3.06* | 21.6±6.05* | 16.9±1.64 | 9±11.1 | *0.016* |
| **stride length left**  (% of Subject’s height) | 70.1±8.39* | 75±5.69 | 73.3±5.99 | 71.5±9.25* | 82.1±4.35 | 8±11.7 | *0.008* |
| **stride length right**  (% of Subject’s height) | 69.8±7.96* | 74.9±5.34 | 73.9±5.74 | 71.4±8.92* | 81.9±4.13 | 8.2±11.7 | *0.006* |
| **stride velocity left**  (% of Subject’s height/s) | 62.3±9.01 | 67.0±8.28 | 65.0±7.21 | 64.0±9.43 | 77.9±6.27 | 8.5±12.0 | *0.001* |
| **stride velocity right**  (% of Subject’s height/s) | 62.2±8.70 | 74.9±5.34 | 65.1±7.02 | 63.9±9.10 | 77.8±6.33 | 22.4±0.17 | *<0.001* |
| **cadence** (steps/min) | 106.9±10.72 | 107.1±9.51 | 106.3±9.17 | 108.2±11.72 | 113.2±7.28 | 0.4±4.3 | 0.99 |
| **gait cycle time** (s) | 1.1±0.13 | 1.1±0.12 | 1.1±0.11 | 1.1±0.13 | 1.1±0.08 | 0.01±4.0 | 0.99 |
| **double support**  (% of the GCT) | 23.0±2.98 | 22.6±3.54 | 23.3±3.56 | 23.2±3.9 | 20.4±3.16 | 1.5±0.7 | 0.91 |
| **swing** (% of the GCT)**/stance** (% of the GCT) **left** | 38.6±1.69 | 38.5±2.12 | 38.3±2.26 | 39.1±2.19 | 39.4±1.85 | 0.1±4.7 | 0.99 |
| **swing** (% of the GCT)**/stance** (% of the GCT) **right** | 38.3±2.24 | 38.7±2.07 | 38.5±2.22 | 37.8±2.08 | 40.1±1.62 | 0.9±4.5 | 0.82 |
| **arm velocity left** (deg/s) | 151.0±73.38 | 157.7±52.57 | 149.7±44.3 | 155.8±74.73 | 195.0±43.3 | 20.5±0.5 | 0.95 |
| **arm velocity right** (deg/s) | 136.7±84.95 | 153.7±55.76 | 149.2±76.95 | 157.6±65.8 | 190.5±52.7 | 33.1±0.5 | 0.63 |
| **arm ROM left** (deg) | 15.1±8.62 | 22.7±11.32 | 21.34±10.67 | 17.6±8.62 | 32.6±13.22 | 82.3±46.6 | *<0.001* |
| **arm ROM rigth** (deg) | 10.2±6.93 | 14.8±9.96 | 11.9±7.56 | 15.9±9.55 | 24.4±12.0 | 58.5±72.01 | *<0.001* |
| **trunk ROM horizontal** (deg) | 4.9±2.56 | 6.0±2.78 | 5.4±2.53 | 5.5±2.56 | 7.1±1.97 | 26.9±25.6 | *<0.001* |
| **trunk ROM sagittal** (deg) | 3.1±0.74 | 3.7±0.9 | 3.3±0.87 | 3.5±0.82 | 4.1±0.91 | 24.1±32.2 | *0.001* |
| **trunk ROM frontal** (deg) | 8.3±3.45 | 9.3±3.69 | 9.1±3.69 | 8.9±3.37 | 11.4±2.68 | 15.1±22.4 | *0.006* |
| **trunk velocity horizontal** (deg/s) | 20.3±6.89 | 24.0±6.81 | 21.7±6.63 | 22.5±6.04 | 30.14±8.65 | 21.6±23.5 | *<0.001* |
| **trunk velocity sagittal** (deg/s) | 17.9±5.40 | 21.7±5.87 | 19.7±7.5 | 20.6±5.45 | 25.5±6.50 | 23.4±4.27 | *<0.001* |
| **trunk velocity frontal** (deg/s) | 32.0±9.78 | 37.6±11.32 | 36.0±10.34 | 34.4±8.85 | 45.3±8.91 | 19.0±17.17 | *<0.001* |
| **turn duration** (s) | 2.9±0.85* | 2.52±1.30* | 2.46±0.61 | 2.63±1.01* | 2.15±0.45 | 13.7±29.3 | 0.23 |
| **turn peak velocity** (deg/s) | 129.4±30.9 | 152.3±36.27 | 150.4±24.7 | 147.7±40.3 | 159.4±25.93 | 19.7±25.93 | *<0.001* |
| **sit to stand duration** (s) | 2.3±0.41 | 2.2±0.37 | 2.2±0.40 | 2.4±0.52 | 2.1±0.31 | 5.4±19.84 | 0.49 |
| **sit to stand velocity** (deg/s) | 79.4±18.55 | 95.6±30.36 | 86.6±26.91 | 91.0±26.64 | 103.3±25.23 | 19.9±21.96 | *<0.001* |
| **sit to stand ROM** (deg) | 30.2±6.67 | 33.3±7.41 | 30.4±7.04 | 31.4±7.16 | 33.8±7.40 | 11.7±16.6 | *0.017* |
| **turn to sit duration** (s) | 4.4±1.16* | 3.9±0.72* | 4.1±0.92* | 4.1±1.18* | 3.5±0.28 | 8.5±14.75 | *0.049* |
| **turn to sit velocity** (deg/s) | 126.5±31.83 | 148.2±37.43 | 140.2±32.21 | 140.0±36.49 | 175.1±21.61 | 19.3±22.62 | *<0.001* |
| **turn to sit ROM** (deg) | 17.6±6.61 | 17.6±6.81 | 17.1±4.81* | 18.0±6.93 | 28.9±9.93 | 9.4±44.35 | 0.99 |

_ITUG: Instrumented Timed Up and Go Test; *p value in comparison with control values; StimON: both-sided STN stimulation ON; OFF: both-sided STN stimulation OFF; L-StimON: left STN stimulation ON; R-StimON: right STN stimulation ON; ROM: range of motion; GCT: gait cycle time_
